# Supplementary material for: Phylomitogenomics elucidates the evolution of symbiosis in Thoracotremata (Decapoda: Cryptochiridae, Pinnotheridae, Varunidae)
Source: PeerJ. 2023 Oct 16;11:e16217. doi: 10.7717/peerj.16217 (PMC10586294; doi:10.7717/peerj.16217)
Supplement: Supplemental Information 1 [file peerj-11-16217-s001.docx]

**Supplementary Materials**

**Phylomitogenomics elucidates the evolution of symbiosis in Thoracotremata (Decapoda: Cryptochiridae, Pinnotheridae, Varunidae)**

Tao Xu ^1^, Henrique Bravo ^1^, Sancia E.T. van der Meij ^1, 2^

^1^ GELIFES, University of Groningen, Groningen, The Netherlands

^2^ Marine Biodiversity Group, Naturalis Biodiversity Center, Leiden, The Netherlands

Corresponding Author:

Tao Xu^1^

Nijenborgh 7, 9747 AG, Groningen, The Netherlands

E-mail address: parksonxt@163.com

**Supplementary Tables**

**Table S7.** Wilcoxon rank sum test between symbiotic and free-living groups in the free-ratio model, and the average ω ratio (excluding the outliers shown in Fig. 4).

| Genes | Wilcoxon rank sum test (P-values) | Free-living  (Average ω ratio) | Symbiotic  (Average ω ratio) | Is ω ratio (free-living >symbiotic) |
| --- | --- | --- | --- | --- |
| *atp6* | 0.04 | 0.0160 | 0.0208 | Yes |
| *atp8* | 0.62 | 0.0415 | 0.0472 | Yes |
| *cob* | 0.04 | 0.0084 | 0.0106 | Yes |
| *cox1* | 0.27 | 0.0057 | 0.0060 | Yes |
| *cox2* | 0.66 | 0.0112 | 0.0095 | No |
| *cox3* | 0.30 | 0.0119 | 0.0112 | No |
| *nad1* | 0.52 | 0.0099 | 0.0104 | Yes |
| *nad2* | 0.42 | 0.0294 | 0.0274 | No |
| *nad3* | 0.10 | 0.0248 | 0.0314 | Yes |
| *nad4* | 0.29 | 0.0182 | 0.0200 | Yes |
| *nad4l* | 0.45 | 0.0193 | 0.0159 | No |
| *nad5* | 0.02 | 0.0164 | 0.0214 | Yes |
| *nad6* | 0.23 | 0.03016 | 0.0330 | Yes |

**Table S8.** The results of selective pressure analysis of each mitochondrial gene based on the branch-site model (Model A null vs Model A) for the gall crabs (Cryptochiridae), pea crabs (Pinnotheridae), and two symbiotic varunids. In the Bayes Empirical Bayes (BEB), one star represents sites with greater posterior probability (PP ≥ 95%), two stars (PP ≥ 99%) are positively selected.

|  | Protein | Model A null (lnL) | Model A (lnL) | df | Chi-square (p) | Positively selected site (BEB: PP≥95%) |
| --- | --- | --- | --- | --- | --- | --- |
| Cryptochiridae | *atp6* | -15128.656897 | -15128.656897 | 1 | 1 |  |
|  | *atp8* | -4589.891381 | -4588.217852 | 1 | 0.067 | 4 M 0.999**  8 F 0.999**  10 L 0.998**  16 F 0.981*  26 E 1.000** |
|  | *cob* | -23324.546950 | -23324.546950 | 1 | 1 | 25 L 0.530  207 S 0.923  276 T 0.969*  321 I 0.658 |
|  | *cox1* | -29862.398315 | -29862.398315 | 1 | 1 |  |
|  | *cox2* | -11900.142779 | -11900.142779 | 1 | 1 |  |
|  | *cox3* | -14890.249110 | -14890.249110 | 1 | 1 |  |
|  | *nad1* | -18850.470119 | -18850.470119 | 1 | 1 |  |
|  | *nad2* | -28428.159441 | -28428.159441 | 1 | 1 |  |
|  | *nad3* | -7663.856629 | -7663.856629 | 1 | 1 |  |
|  | *nad4* | -28653.448974 | -28653.448974 | 1 | 1 |  |
|  | *nad4l* | -5739.354145 | -5739.354145 | 1 | 1 |  |
|  | *nad5* | -33283.990859 | -33283.990859 | 1 | 1 | 5 F 0.976*  13 V 0.701  14 V 0.594  25 S 0.689  350 I 0.518  354 M 0.998**  400 V 0.825  404 V 0.617 |
|  | *nad6* | -12872.787310 | -12957.575664 | 1 | 0 | 63 F 0.999**  78 L 0.589  79 T 0.877  81 S 0.929  89 P 1.000** |
| Pinnotheridae | *atp6* | -16057.911731 | -16057.911731 | 1 | 1 |  |
|  | *atp8* | -4883.178500 | -4883.178500 | 1 | 1 | 26 S 0.955*  28 I 0.904 |
|  | *cob* | -24752.871004 | -24752.871004 | 1 | 1 | 310 D 0.999** |
|  | *cox1* | -31822.239128 | -31822.239128 | 1 | 1 |  |
|  | *cox2* | -12797.444979 | -12797.444979 | 1 | 1 |  |
|  | *cox3* | -16116.700486 | -16116.700486 | 1 | 1 |  |
|  | *nad1* | -19947.072622 | -19947.072622 | 1 | 1 |  |
|  | *nad2* | -30077.074307 | -30077.074307 | 1 | 1 | 50 S 0.976*  55 I 0.988*  106 V 0.843 |
|  | *nad3* | -8263.625226 | -8263.625226 | 1 | 1 |  |
|  | *nad4* | -30340.873416 | -30340.873416 | 1 | 1 | 125 L 0.995**  126 V 0.993**  130 G 0.938  131 V 1.000**  318 S 0.913 |
|  | *nad4l* | -6087.498550 | -6087.498550 | 1 | 1 |  |
|  | *nad5* | -35214.671347 | -35214.671347 | 1 | 1 | 149 L 0.521  158 S 1.000**  214 A 0.998**  215 S 0.843  371 S 0.890  400 T 0.947  426 I 1.000** |
|  | *nad6* | -13673.134214 | -13673.134214 | 1 | 1 | 35 F 0.999**  71 S 0.951*  75 F 1.000**  80 T 0.692  82 P 0.882  91 N 0.714 |
| *A. inaequipes* and *T. horvathi* (Varunidae) | *atp6* | -14377.525391 | -14377.288921 | 1 | 0.492 |  |
|  | *atp8* | -4322.020065 | -4320.835179 | 1 | 0.124 | 10 L 0.883  18 L 0.943  26 N 0.999** |
|  | *cob* | -22528.738913 | -22528.738913 | 1 | 1 |  |
|  | *cox1* | -28799.697975 | -28799.697975 | 1 | 1 |  |
|  | *cox2* | -11377.588529 | -11377.588529 | 1 | 1 |  |
|  | *cox3* | -14347.446919 | -14346.507202 | 1 | 0.170 |  |
|  | *nad1* | -18111.752616 | -18111.445638 | 1 | 0.433 |  |
|  | *nad2* | -27172.619495 | -27172.619495 | 1 | 1 |  |
|  | *nad3* | -7374.039532 | -7374.039532 | 1 | 1 |  |
|  | *nad4* | -27452.136684 | -27452.136684 | 1 | 1 |  |
|  | *nad4l* | -5427.607573 | -5427.607573 | 1 | 1 |  |
|  | *nad5* | -31973.682602 | -31973.408566 | 1 | 0.459 | 4 G 0.723  21 A 0.909  37 L 0.602  112 S 0.997**  380 V 0.869  404 C 0.993**  423 S 0.893  436 I 0.925 |
|  | *nad6* | -12434.494402 | -12434.494402 | 1 | 1 |  |

Note: When the evidence for positive selection is not very strong, the Likelihood-Ratio Test (LRT) and BEB may show conflicting results. In particular, when p>0.05, BEB can still detect several positive selection sites (i.e. genes *atp8*, *cob and nad5* in Cryptochiridae; genes *atp8*, *nad2*, *nad4*, *nad5* and *nad6* in Pinnotheridae; genes *atp8* and *nad5* in symbiotic Varunidae*.*).

**Supplementary Figures**

**Figure S1.** The Relative Synonymous Code Usage (RSCU) for concatenated Protein Coding Genes (PCGs) of: A) *Kroppcarcinus siderastreicola*; B) *Opecarcinus hypostegus*; C) *Troglocarcinus corallicola*; and D) *Hapalocarcinus marsupialis* s.l..

**Figure S2.** Secondary structures of 22 transfer RNA genes of: A) *Kroppcarcinus siderastreicola*; B) *Opecarcinus hypostegus*; C) *Troglocarcinus corallicola*; and D) *Hapalocarcinus marsupialis* s.l.. The names of the mitochondrial transfer RNA genes can be found in Table S2.

**Figure S3.** Bayesian inference tree based on a concatenated dataset including 13 Protein Coding Genes (PCGs) and two ribosomal RNA genes (16s rRNA and 12s rRNA) by MrBayes.
