# Supplementary figures and images for: Phylomitogenomics elucidates the evolution of symbiosis in Thoracotremata (Decapoda: Cryptochiridae, Pinnotheridae, Varunidae)
Source: PeerJ. 2023 Oct 16;11:e16217. doi: 10.7717/peerj.16217 (PMC10586294; doi:10.7717/peerj.16217)

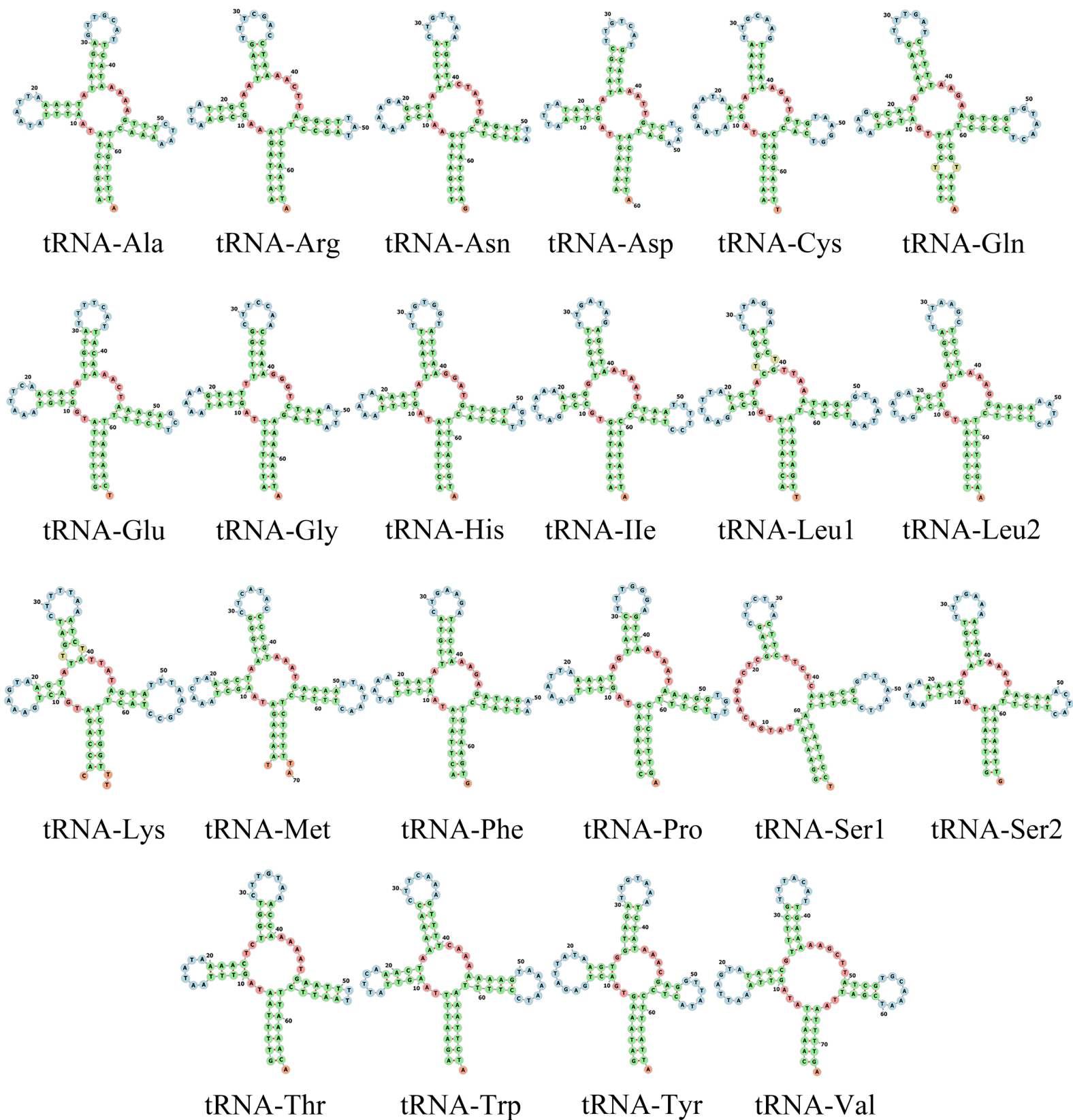

A) Secondary structures of 22 transfer RNA genes in *Troglocarcinus corallicola*

Supplement: Figure S2A [file peerj-11-16217-s004.pdf]

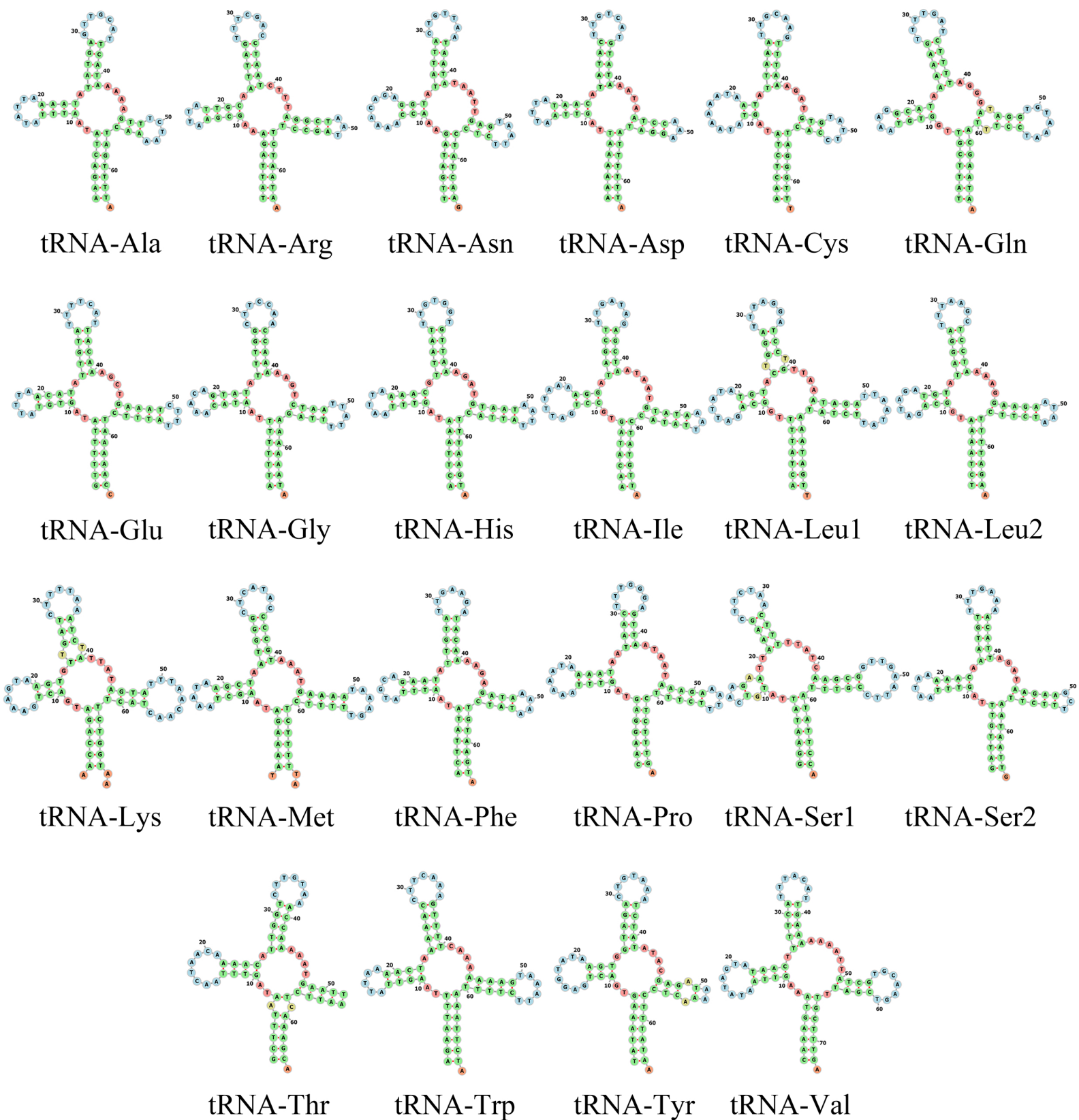

B) Secondary structures of 22 transfer RNA genes in *Kroppcarcinus siderastreicola*

Supplement: Figure S2B [file peerj-11-16217-s005.pdf]

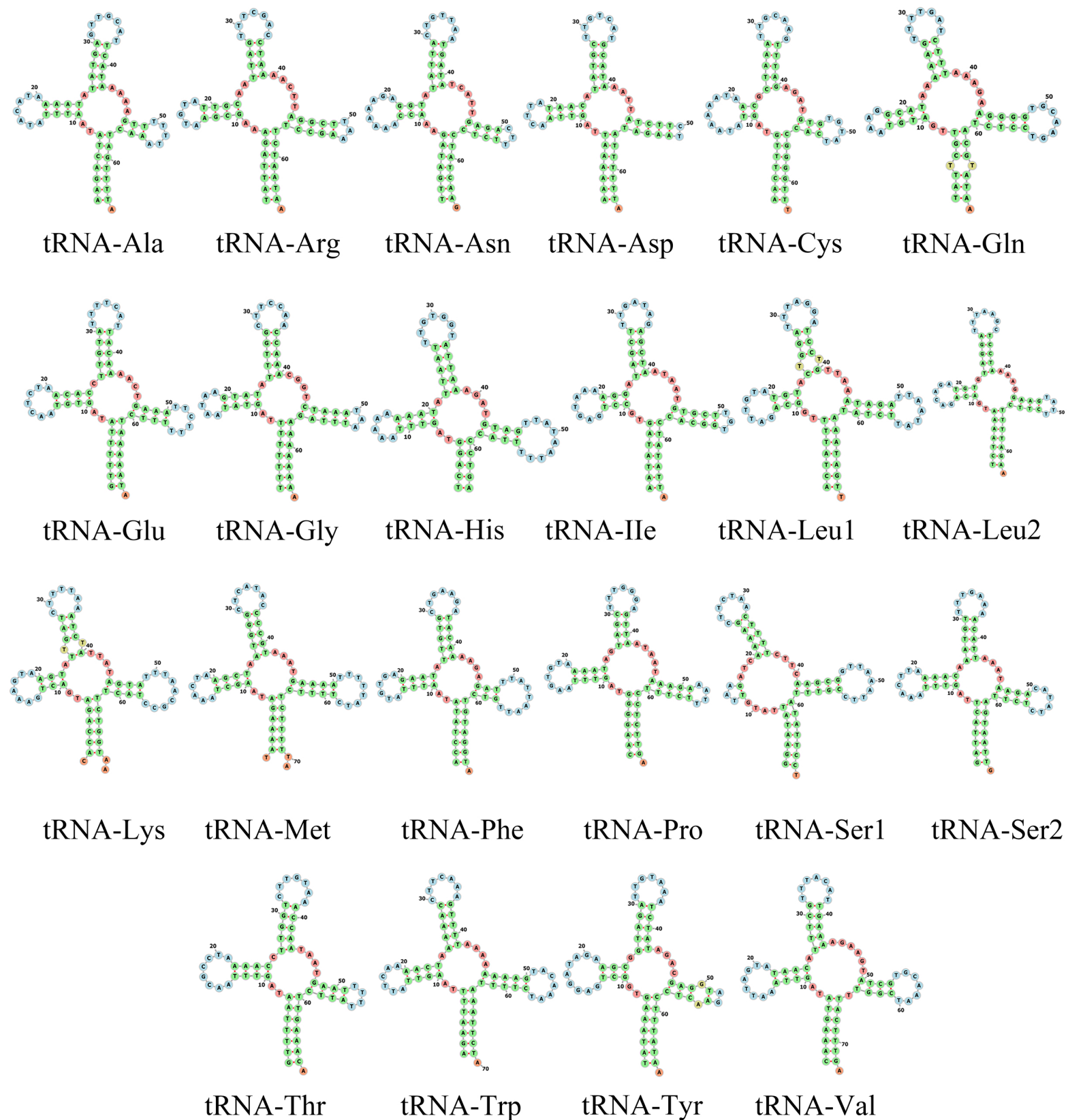

C) Secondary structures of 22 transfer RNA genes in *Opecarcinus hypostegus*

Supplement: Figure S2C [file peerj-11-16217-s006.pdf]

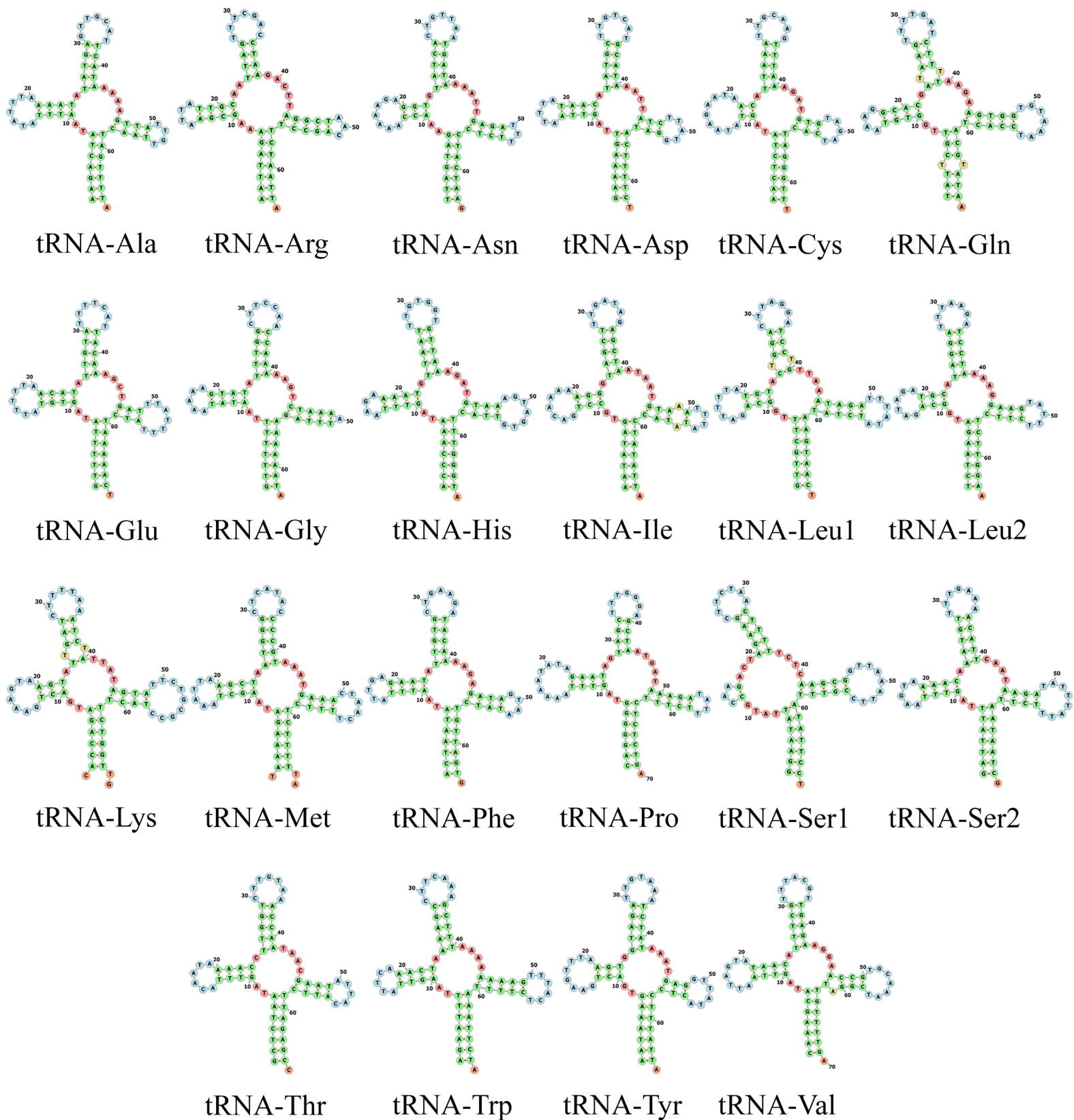

D) Secondary structures of 22 transfer RNA genes in *Hapalocarcinus marsupialis* s.l.

Supplement: Figure S2D [file peerj-11-16217-s007.pdf]

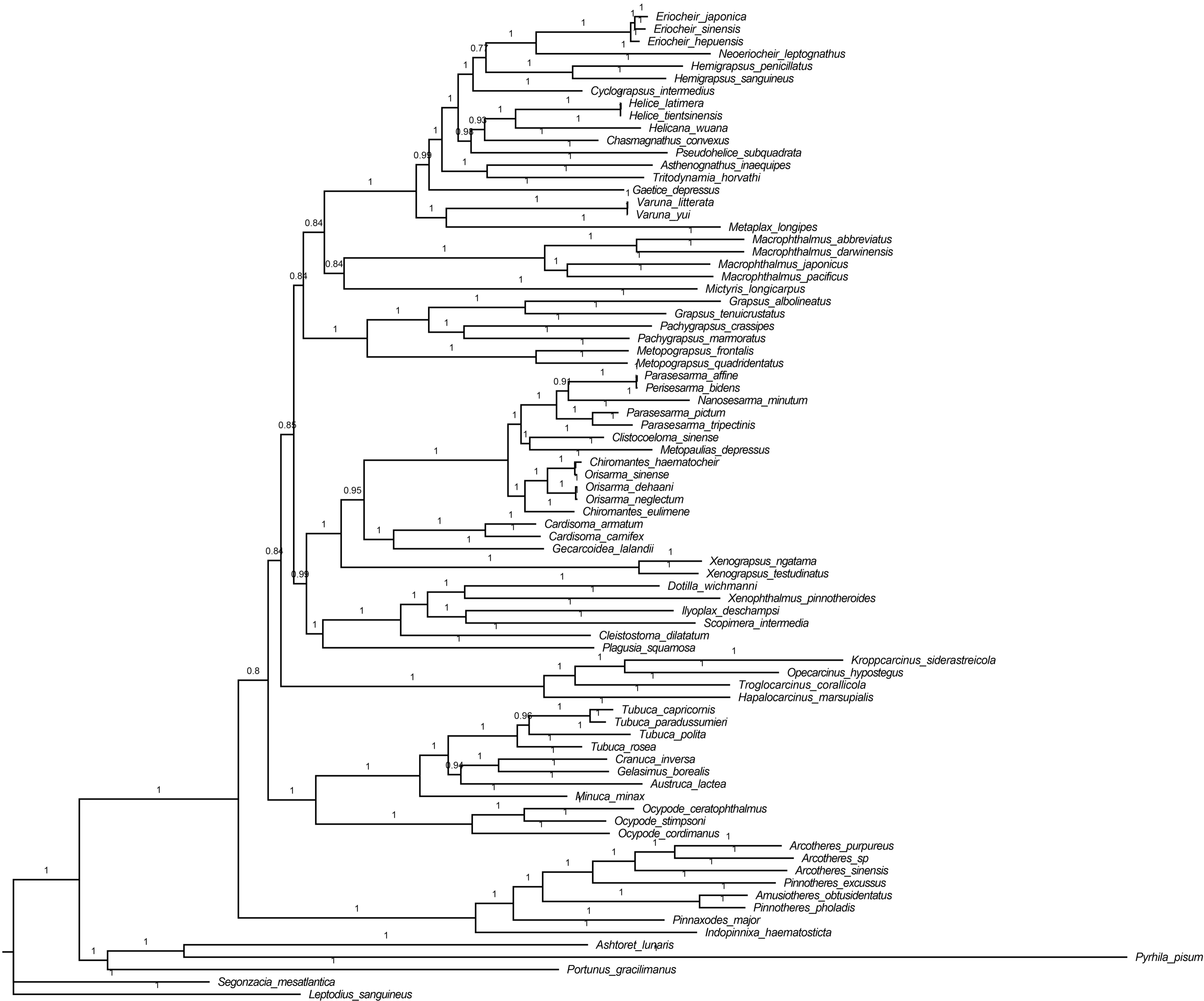

0.3

Supplement: Figure S3 [file peerj-11-16217-s008.pdf]
