# Supplementary material for: Phylomitogenomics elucidates the evolution of symbiosis in Thoracotremata (Decapoda: Cryptochiridae, Pinnotheridae, Varunidae)
Source: PeerJ. 2023 Oct 16;11:e16217. doi: 10.7717/peerj.16217 (PMC10586294; doi:10.7717/peerj.16217)
Supplement: Figure S1 — (A) Kroppcarcinus siderastreicola; (B) Opecarcinus hypostegus; (C) Troglocarcinus corallicola; and (D) Hapalocarcinus marsupialis s.l. [file peerj-11-16217-s003.pdf]

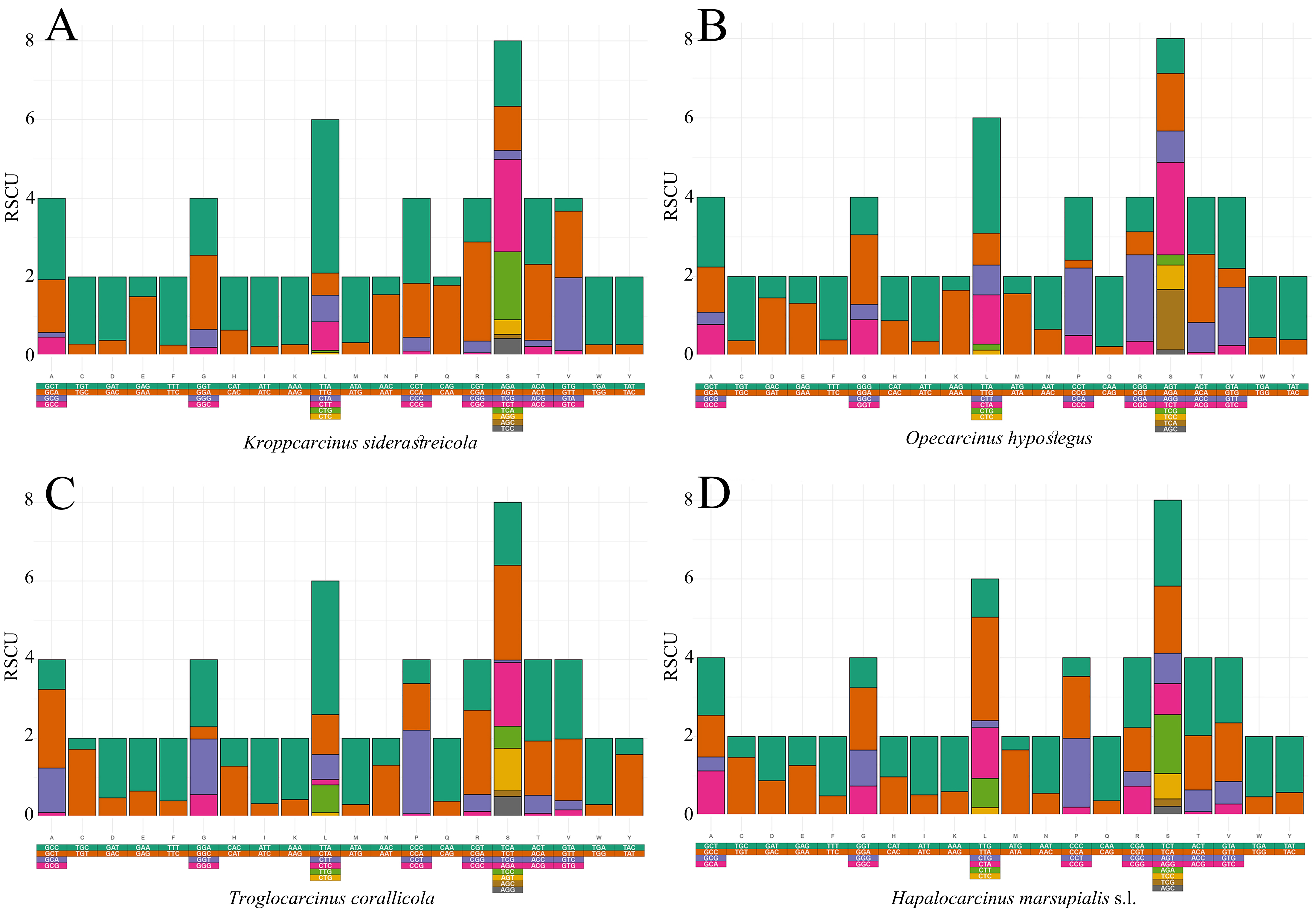
A
B
C
D
RSCU
0
2
4
6
8
A C D E F G H I K L M N P Q R S T V W Y
Kroppcarcinus siderastreicola
Opecarcinus hypostegus
Troglocarcinus corallicola
Hapalocarcinus marsupialis s.l.
